# The Practice of Surgery

**Published:** 1857-12

**Authors:** 


*The Practice of Surgery.* By James Miller, F. R. S. C., F. R. C. S. E.' etc., etc. Revised by the American Editor. Fouath American from the last Edinburgh Edition.

We see by the publisher's notice, that the present edition was superintended by a professional gentleman of Philadelphia, owing to the absence of Dr. Sargent in Europe. The American editor, however, made use of Dr. Sargent's notes in its preparation.

This is indeed an admirable work, and such an one as we ought to have from some of our American surgeons. There is a deficiency still in our national surgical literature, which we hope and expect will be filled by Prof. Gross, whose work on surgery is rapidly advancing. It is true that one of our countrymen has lately published a system of surgery, but the medical press has pretty well decided how far that work goes to fill up the hiatus. We are still compelled to use works by foreign authors in this department, among which Miller's Surgery ranks high.

The present work really forms a second volume to Miller's Principles, they together forming a complete system of surgery. F.

